# Lifetime of Ionic Vacancy Created in Redox Electrode Reaction Measured by Cyclotron MHD Electrode

**DOI:** 10.1038/srep19795

**Published:** 2016-01-21

**Authors:** Atsushi Sugiyama, Ryoichi Morimoto, Tetsuya Osaka, Iwao Mogi, Miki Asanuma, Makoto Miura, Yoshinobu Oshikiri, Yusuke Yamauchi, Ryoichi Aogaki

**Affiliations:** 1 Research Organization for Nano and Life Innovation, Waseda University, Shinjuku-ku, Tokyo 162-0041, Japan; 2 Saitama Prefectural Showa Water Filtration Plant, Kasukabe, Saitama 344-0113, Japan; 3 School of Science and Engineering, Waseda University, Shinjuku-ku, Tokyo 169-8555, Japan; 4 Institute for Materials Research, Tohoku University, Sendai, Sendai 980-8577, Japan; 5 Yokohama Harbor Polytechnic College, Naka-ku, Yokohama 231-0811, Japan; 6 Hokkaido Polytechnic College, Otaru, Hokkaido 047-0292, Japan; 7 Yamagata College of Industry and Technology, Matsuei, Yamagata 990-2473, Japan; 8 National Institute for Materials Science, Tsukuba, Ibaraki 305-0044, Japan; 9 Polytechnic University, Sumida-ku, Tokyo 130-0026, Japan

## Abstract

The lifetimes of ionic vacancies created in ferricyanide-ferrocyanide redox reaction have been first measured by means of cyclotron magnetohydrodynamic electrode, which is composed of coaxial cylinders partly exposed as electrodes and placed vertically in an electrolytic solution under a vertical magnetic field, so that induced Lorentz force makes ionic vacancies circulate together with the solution along the circumferences. At low magnetic fields, due to low velocities, ionic vacancies once created become extinct on the way of returning, whereas at high magnetic fields, in enhanced velocities, they can come back to their initial birthplaces. Detecting the difference between these two states, we can measure the lifetime of ionic vacancy. As a result, the lifetimes of ionic vacancies created in the oxidation and reduction are the same, and the intrinsic lifetime is 1.25 s, and the formation time of nanobubble from the collision of ionic vacancies is 6.5 ms.

Hydrated electron is a key reactive intermediate in the chemistry of water, including the biological effects of radiation. In water, a cavity takes a quasi-spherical shape with a 2.5 Å radius surrounded by at least six OH bonds oriented toward the negative charge distribution where an equilibrated hydrated electron is transiently confined^1^,^2^. Though stabilized by the cavity, as have been criticized by Bockris and Conway concerning hydrated electron in cathodic hydrogen evolution^3^, the electron has quite short lifetimes of the order of 100 femtoseconds.

Recently, it has been newly found that a quite different type of cavity, i.e., ionic vacancy in aqueous electrolyte is produced by electrode reactions. Ionic vacancy is a popular point defect in solid electrolytes^4^,^5^,^6^,^7^. In liquid electrolyte solutions, for a long time, its stable formation has been regarded impossible. However, in recent years, it has been clarified that ionic vacancies are stoichiometrically created in electrode reactions^8^, and easily converted to nanobubbles^9^. Ionic vacancy in liquid solution is an electrically polarized free vacuum void with a 0.1 nm order diameter surrounded by oppositely charged ionic cloud. As a result, its direct observation is quite hard, but possible after nanobubble formation^10^,^11^,^12^,^13^,^14^,^15^,^16^,^17^. For the nanobubble formation and its detection, magnetoelectrochemistry can provide a useful tool. In magnetically assisted electrolysis under a magnetic field parallel to electrode surface, Lorentz force induces a solution flow called magnetohydrodynamic (MHD) flow enhancing mass transport of ions^18^. The fluid flow often yields surface waves and stationary vortexes, which also promotes the convective motion. A theoretical prediction by Fahidy^19^ for aqueous electrolytes was corroborated by experimental evidence^20^ produced in a concentric cylindrical cell using copper electrodes and aqueous cupric sulfate electrolytes. For studying the mass transport in the MHD flow, MHD impedance technique has been developed by Olivier *et al.*^21^,^22^, which is based on the frequency response of limiting currents observed in the presence of sinusoidally excited magnetic fields. An application of the MHD flow in a parallel magnetic field led to the development of MHD-pumping electrode cells called MHD electrode (Aogaki *et al.*)^23^, where the concentration distribution, modeled by the classical convective diffusion equation, reduces to the simple form of the limiting diffusion current *i*_L_ = *kB*^1/3^, where *k* is a constant, and *B* is the applied magnetic flux density. In a viscous flow in a narrow channel^24^, *i*_L_ is proportional to *B*^1/2^. In both pumping-cell configurations, agreement between theory and experimental results is excellent. These results therefore show a notable advantage of magnetically excited solution flow lying in the practical possibility of using very small cells without mechanical means. Under a vertical magnetic field, as shown in Fig. 1, a macroscopic tornado-like rotation of the solution called vertical MHD flow is formed on an electrode surface^25^. Inside the rotation, numerous minute vortexes called micro-MHD flows are generated, which, in electrodeposition, yields a deposit with chiral structure^26^. Mogi has first found that by using electrodes fabricated in the same way, chiral selectivity appears in enantiomorphic electrochemical reactions^27^,^28^. At the same time, in a vertical MHD flow, the collision of ionic vacancies is strongly promoted, so that the conversion of ionic vacancies to nanobubbles is accelerated. The nanobubbles once evolved are quickly gathered to form microbubbles. Figure 2 exhibits photos of micro-bubble evolution in ferricyanide-ferrocyanide redox reaction without any electrochemical gas evolution^29^. After this report, the same kinds of photos of micro-bubble evolution have been taken in copper cathodic deposition^30^ and copper anodic dissolution^31^. These experimental results obviously inform us that ionic vacancy is a sub-product, generally created in electrode reaction. As a result, next question is opened to us, i.e., how is the lifetime of ionic vacancy? As mentioned initially, it has been believed that in electrolyte solutions, if their existence were possible, the lifetimes would be infinitesimally short. In the present paper, therefore, in one of the most basic electrode reactions, i.e., ferricyanide-ferrocyanide redox reaction, the lifetimes of ionic vacancies are measured by a cyclotron magnetohydrodynamic electrode (CMHDE), which is composed of a pair of coaxial cylinders equipped with partly exposed electrodes in a vertical magnetic field.

## Theory

A CMHDE is, as shown in Fig. 3, composed of two concentric cylindrical electrodes acting as working (WE) and counter (CE) electrodes, which are, partly exposed, forming a pair of arc surfaces with the same open angles. In a magnetic field parallel to the axis of the cylinders, electrolytic current flows between the electrodes, so that Lorentz force induced moves the solution along the circumferences of the cylinders. Along the electrodes, ionic vacancies created proceed with the solution from the electrode surface to the adjacent insulated wall. Under a low magnetic field, the vacancies become extinct according to their lifetimes, so that the friction of rigid surfaces controls the solution flow. However, under a high magnetic field, due to enhanced fluid motion, they can return to their initial birthplaces, covering the whole surface of the walls. Owing to iso-entropic property^8^, the vacancies act as atomic-scale lubricant, so that the wall surfaces are changed from rigid with friction to free without friction. At the same time, the solution velocity is also changed from rigid mode to free mode, so that the electrolytic current of the free mode behaves in a different way from that of the rigid mode.

### The velocity distribution

For the fluid motion in a cylindrical channel of CMHDE, the Navier-Stokes equation in a cylindrical polar coordinate system (*r*, *ϕ*, *z*) is used. Assuming uniform velocity distribution in the axial (*z*-axis) direction and magnetic field applied in the axial direction, we obtain the equations of the radial and transverse velocities. In the present case, due to low electric conductivity of liquid electrolyte solution, any electromagnetic induction is disregarded.


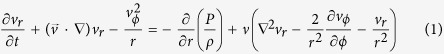



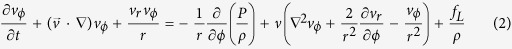


where *v*_*r*_ and *v*_*ϕ*_ are the radial and transverse components of the velocity 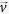, respectively. *f*_*L*_ is the Lorentz force per unit volume, *P* is the pressure, *ρ* is the bulk density, and *ν* is the kinemtic viscosity. *f*_*L*_ is defined by


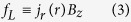


where *B*_*z*_ is the magnetic flux density in the *z*-direction, and *j*_*r*_(*r*) is the radial current density. Equations 1 and 2 allow us to derive the steady-state solution of the following forms


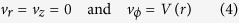


Equation 1 describes the effect of a centrifugal force, which, in a small-scale situation such as the present case, can be neglected. On the other hand, Eq. 2 can be solved under small Reynolds number for a viscid flow, i.e., *Re* ≪ 1 as follows: in view of axisymmetry together with the above condition, Eq. 2 is averaged with regard to *ϕ* from 0 to 2*π* by the following integration in steady state.


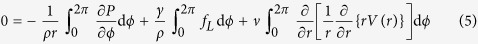


where *γ* is the cell constant, which is introduced by the conversion efficiency of the work by the Lorentz force *f*_*L*_ to the kinetic energy of the circular motion. Considering the relations


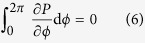


and


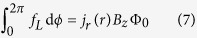


we have


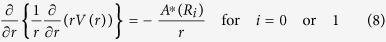


where A^*^(*R*_*i*_) is the Lorentz force factor, depending on which electrode is employed as WE, and *i* = 0 and 1 imply the radii of the inner and outer cylinders, respectively. In Eq. 8, *V*(*r*) is solved for a laminar flow under the condition of *Re* ≪1, which conventionally provides viscid mode on the rigid surfaces with friction. In the present case, however, due to the lubricant nature of ionic vacancy, except for the viscid mode, transient-inviscid mode on the free surfaces without friction newly emerge. Equation 8 is integrated with regard to *r*, so that the transverse velocities for both cases are obtained,


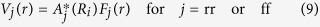


where *j* = rr and ff correspond to the cases of two rigid and two free surfaces of the concentric walls, respectively. The Lorentz force factor A^*^(*R*_*i*_) is defined by


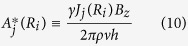


where *J*_*j*_(*R*_*i*_) and 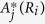 is the total current *J*(*R*_*i*_) and the Lorentz force factor A^*^(*R*_*i*_) for *j* = rr or ff. *F*_*j*_(*r*) is the geometric factor defined by


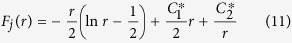


where 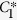 and 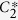 are arbitrary constants, which are determined by the boundary conditions of the rigid and free surfaces.

To calculate the mass transfer in the diffusion layer, the velocity distribution near the electrode surface must be provided, which is obtained by the first expansion of the velocity at the working electrode.


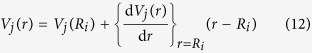


#### a) Viscid flow on two rigid surfaces

For rigid surfaces, under the boundary conditions,


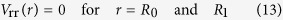


the velocity near the electrode surface at *r* = *R*_*i*_ is obtained as


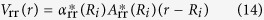


where 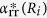 is the surface factor of viscid flow, i.e.,


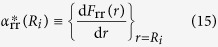


The surface factor 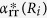 are expressed by


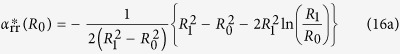


and


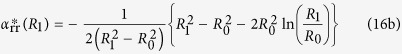


#### b) Transient inviscid flow on two free surfaces

For free surfaces without friction, under the boundary conditions


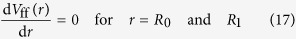


the velocity near the electrode surface at *r* = *R*_*i*_ is expressed by


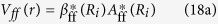



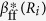
 is the surface factor of transient inviscid mode, i.e.,


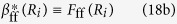


Equation 18a expresses a piston flow independent of the radial coordinate. The surface factor 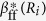 are expressed by


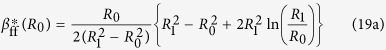


and


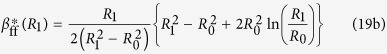


### The diffusion current equations

As shown in Fig. 4, a diffusion layer is formed in accordance with electrode reaction^32^. To analyze the mass transfer process, a concentric arc element 1243 with an infinitesimal angle of d*ϕ* is introduced. The amount of the reactant carried by the fluid through the plane 12 per unit time is 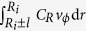, where *C*_*R*_ is the reactant concentration, *l* is the distance chosen greater than the diffusion layer thickness *δ*_*c*_, and the sign ± corresponds to *i* = 0 (inner WE) and 1 (outer WE), respectively.

Using the mass transfer equations in viscid and transient-inviscid flows, we derive the steady state currents in viscid and transient-inviscid modes in the following:

#### a) The current in a viscid flow

According to Eq. A.8 in Appendix A, the steady-state mass transfer equation for a viscid flow is expressed by


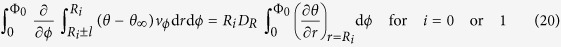


where *i* = 0 and 1 implies that WE is located at inner and outer cylinders, respectively. For simplicity, the concentrations of the surface *C*_*R*_(*R*_*i*_) and the bulk *C*_*R*_(∞) are converted to


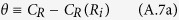



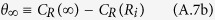


The boundary conditions of *θ* are as follows,


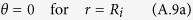



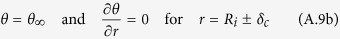


The simplest function form of *θ* satisfying the boundary conditions Eqs A.9 a and A.9 b is


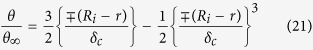


where the sign 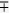 corresponds to *i* = 0 and 1, respectively. The diffusion layer thickness *δ*_*c*_ develops in the transverse direction, which is, according to Levich^33^, expressed by


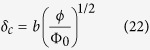


where *b* is the normalized thickness of the diffusion layer. In view of *v*_*ϕ*_ = *V*_*rr*_(*r*), from Eqs 14 and 22, we have


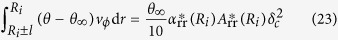


Substituting Eq. 21 into Eq. 23, we can obtain


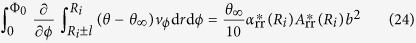


Then, using Eqs 21 and 22, we can perform the following calculation,


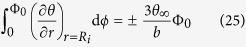


where the sign ± corresponds to *i* = 0 and 1, respectively. Substituting Eqs 24 and 25 into Eq. 20, we obtain


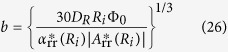


For simplicity, apart from electrochemical definition, i.e., anodic or cathodic, the current density of WE is defined positive.


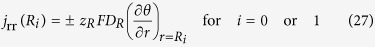


where the sign ± is introduced for the positive current density. *z*_R_ is the charge number, *F* is Faraday constant, and *D*_R_ is the diffusion coefficient.

The average value of Eq. 27 is given by


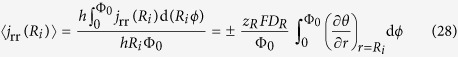


Therefore, substituting Eq. 25 into Eq. 28, and multiplying 〈*j*_rr_(*R*_*i*_)〉 by *R*_*i*_Φ_0_*h*, we obtain the total current


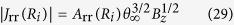


where *A*_rr_(*R*_*i*_) is the current coefficient, defined by


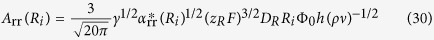


#### b) The current in a transient-inviscid flow

**Figure 1:**
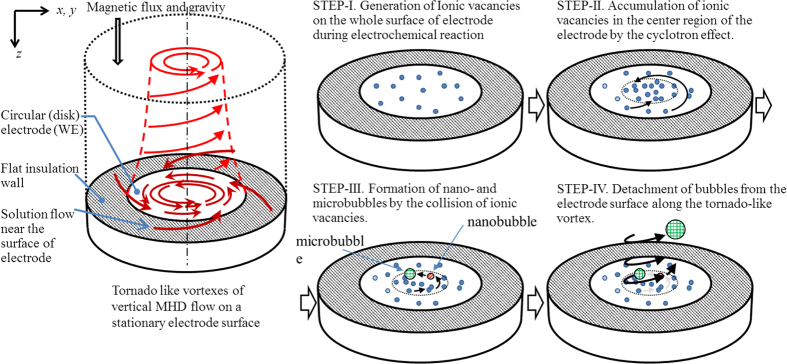
Schematic of vertical MHD flow generated on electrode surface and nano- and micro-bubble formation steps.

**Figure 3:**
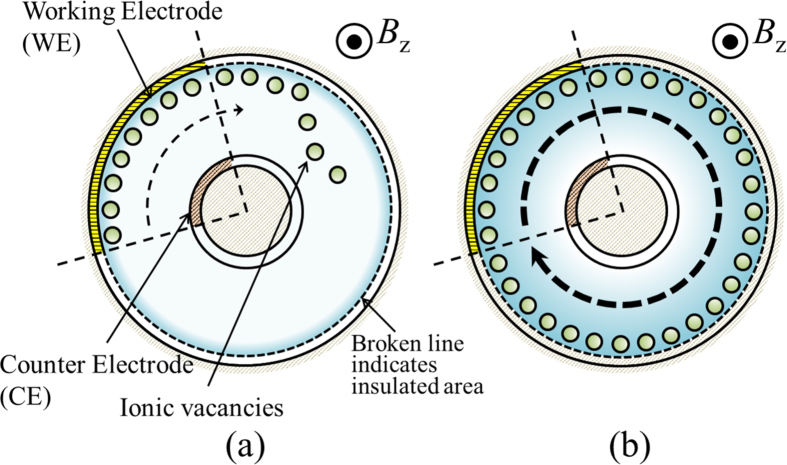
Top views of the circulation of ionic vacancies with the solution flow in the case of reduction in a CMHDE of *i* = 1. (**a**) the case when ionic vacancies vanish (viscid flow), (**b**) the case when ionic vacancies survive (transient-inviscid flow).

In a high magnetic field, due to enhanced velocity, the cylindrical walls are covered with ionic vacancies of lubricant nature, so that the piston flow shown in Eq. 18a emerges. In the same way as vertical MHD flow shown in Fig. 1, microscopic vortexes called micro-MHD flows are induced to assist the mass transfer in the diffusion layer, which is therefore controlled by the piston flow. Introducing the mixing coefficient *ε* by the micro-MHD flows, we can describe the mass transfer equation. In steady state. Eq. A.8 is rewritten by


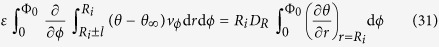


In view of axisymmetry, Eq. 31 is reduced to


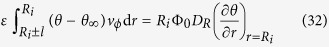


Differently from Eq. 22, in this case, *δ*_*c*_ is a constant with regard to *ϕ*. Since the boundary conditions are the same as Eqs A.9 a and A.9 b, Eq. 21 is also used. The function form of *v*_*ϕ*_ is expressed by Eq. 18a. Therefore, we have


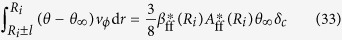


Substituting Eqs 21 and 33 into Eq. 32, we obtain


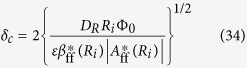


According to Eq. 29, the total current density is obtained.


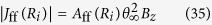


where the current coefficient *A*_ff_(*R*_*i*_) is defined by


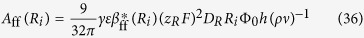


As shown in Eq. 29 and Eq. 35, the total currents observed behave in different ways against magnetic flux density; in the rigid mode, it follows the 1/2nd power of magnetic flux density, whereas in the free mode, it is proportional to the 1st power of magnetic flux density.

#### Measurement of lifetime

The lifetime of ionic vacancy is obtained from the transition of the current from the rigid mode to the free mode, i.e., the two kinds of plot of the current against magnetic flux density provide the point of intersection, which gives rise to the critical magnetic flux density *B*_*zcr*_ together with the critical current *J*_*cr*_. The lifetime is then obtained by


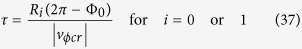


where *v*_*ϕcr*_ is the critical velocity of the MHD flow in the free mode at the free surface, i.e.,


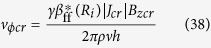


## Results

To calculate the lifetime of ionic vacancy, as shown in Fig. 5, log-log plots of the current vs. magnetic flux density were carried out. As discussed above, from the point of intersection, the critical current *J*_*cr*_ and the critical magnetic flux density *B*_*zcr*_ were obtained. Due to natural convection, in the region of low magnetic field, the current is kept constant. However, as magnetic flux density increases, the current tends to follow a line with a slope of 1/2. By means of Eq. 30, the cell constant *γ* is calculated. Then, according to Eq. 36, the mixing coefficient *ε* is calculated from the plot with a slope of 1 in the region of high magnetic field, which takes a value of the order of 0.01.

The cell constant is, as shown in Eq. 5, defined as the conversion efficiency of the work of Lorentz force to the kinetic energy of MHD flow, i.e.,


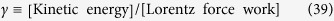


As mentioned above, the original MHD flow is a perfect laminar flow without any disturbances such as vortex flow, so that the streamlines draw concentric, closed loci. If the lifetime of ionic vacancy is sufficiently long, a vacancy once created will continue to move along the same streamline. As a result, the continuous vacancy creation on the electrode inevitably gives rise to the collision of created vacancies with returning vacancies and as formerly predicted^32^, the resultant conversion to nanobubbles. Consequently, in the case of *γ* = 0, i.e., in the absence of fluid flow, ionic vacancies exist without collision, whereas in the case of *γ* = 1, i.e., in the perfect laminar flow, the collision occurs in a 100% probability. This means that the cell constant represents the collision efficiency between created and returning vacancies. As have been discussed above, the cell constant *γ* is not kept constant, but dependent on the electrode configuration such as the electrode height *h* and the angle Φ_0_ of the arc electrode surfaces. This suggests that using a CMHDE, we can perform the collision experiment of ionic vacancies at an efficiency given by cell constant. The ultimate cases of *γ* = 0 and *γ* = 1 correspond to the collisions at probabilities of 0% and 100%, respectively. In accordance with this discussion, in Fig. 6, the lifetimes are plotted against the cell constant in semi-log plot. Whether oxidation or reduction is, all the data form a straight line; the lifetime decreases from the order of 1 s to the order of 1 ms with *γ*. Namely, the vacancies created in the oxidation and reduction have the same lifetimes, and the lifetimes decrease with increasing collision efficiencies. As for the lifetime of ionic vacancy, the following two processes are considered; one is the decay of ionic vacancies to the initial state, and the other is the conversion of ionic vacancies to nanobubbles. As a result, it can be said that the lifetime measured for *γ* = 0 is the intrinsic lifetime of ionic vacancy, whereas the lifetime for *γ* = 1 indicates the formation time of nanobubble via. the collision and coalescence of ionic vacancies. From these discussions, it is concluded that the intrinsic lifetime of the vacancy is 1.25 s, and the formation time of nanobubble is 6.5 ms.

In summary, the lifetimes of ionic vacancies created in ferrocyanide oxdation and ferricyanide reduction are the same, and widely changes from the order of 1 s to the order of 1 ms with the cell constant *γ*, i.e., collision efficiency between ionic vacancies. Namely, by means of CMHDE, the collision process of ionic vacancies in a solution can be analyzed. Based on the nanobubble-formation theory^3^, in the present case, nanobubbles arise from ionic vacancies via. collision and coalescence, and the formation time of nanobubble was derived as 6.5 ms. On the other hand, the intrinsic lifetime of ionic vacancy without collision in this case was determined as 1.25 s.

## Methods

Experiments were performed for ferricyanide-ferrocyanide redox reaction by using a platinum CMHDE. The configuration of the apparatus is shown in Fig. 7. The radii of the inner and outer platinum cylinders were *R*_0_ = 2.0 mm and *R*_1_ = 4.6 mm, respectively, and the angle of the arc electrodes Φ_0_ was changed between 0.2*π* and *π*. The outer and inner electrodes were used as WE and CE, respectively, of which heights were changed between 5 mm and 15 mm for various cell constants to evolve. The whole coaxial cylinders were completely dipped into the solution. A saturated calomel electrode (SCE) was used as reference electrode. To prevent hydrogen and oxygen adsorption and evolution, electrolysis was carried out in limiting-diffusion area at overpotential of ±200 mV (the reduction potential *E*_*red*_ = 30 mV vs. SCE, the oxidation potential *E*_*ox*_ = 430 mV vs. SCE), of which electrode potentials are much more anodic than hydrogen evolution potential and much more cathodic than oxygen evolution potential. Then, to protect the gas evolutions from counter electrode, in view of the difference between the areas of WE and CE, the concentrations of the reactants at CE were chosen three times higher than those of the reactants at WE. The whole apparatus was settled in the bore space (with an upward-oriented magnetic field) of the 40 T superconducting magnet at the high magnetic field center, NIMS, Tsukuba Japan or the 18 T cryocooled superconducting magnet at the High Field Laboratory for Superconducting Materials, IMR, Tohoku University. Temperature of the bore space was kept at 13 °C.

### Appendix A. Mass transfer equation in a viscid flow

**Figure 4:**
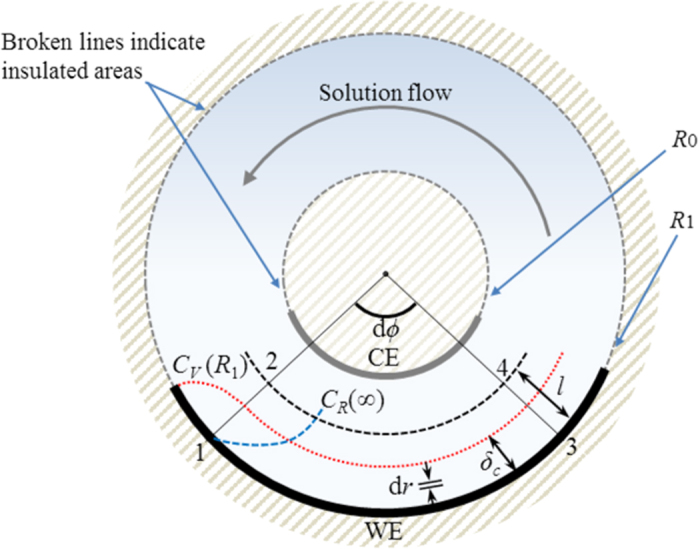
Diffusion layer on the outer WE in viscid mode^32^. *C*_*R*_(∞); the bulk concentration of reactant, *C*_*v*_(*R*_1_); the surface concentration of the ionic vacancy, *δ*_*c*_; the diffusion layer thickness, *l*; the distance chosen greater than *δ*_*c*_, d*ϕ*; the arc angle. *R*_0_; the inner radius, *R*_1_; the outer radius.

**Figure 5:**
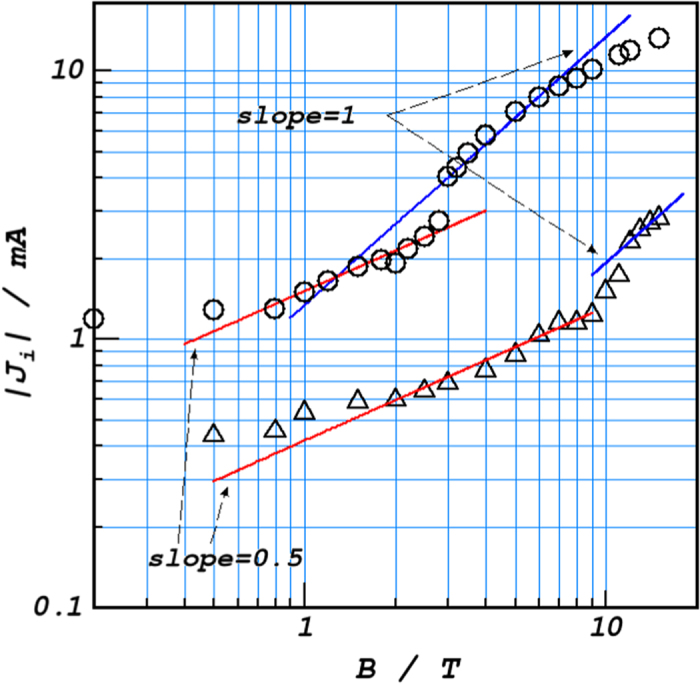
Current vs. magnetic flux density for ferrocyanide/ferricyanide redox reaction in 10^3^_ _mol m^−3^ KCl solutions. o; oxidation of 50 mol m^−3^ ferrocyanide, Δ; reduction of 20 mol m^−3^ ferricyanide. Φ_0_ = *π*. Overpotentials of the oxidation and reduxtion are +200 mV and −200 mV, respectively.

**Figure 6:**
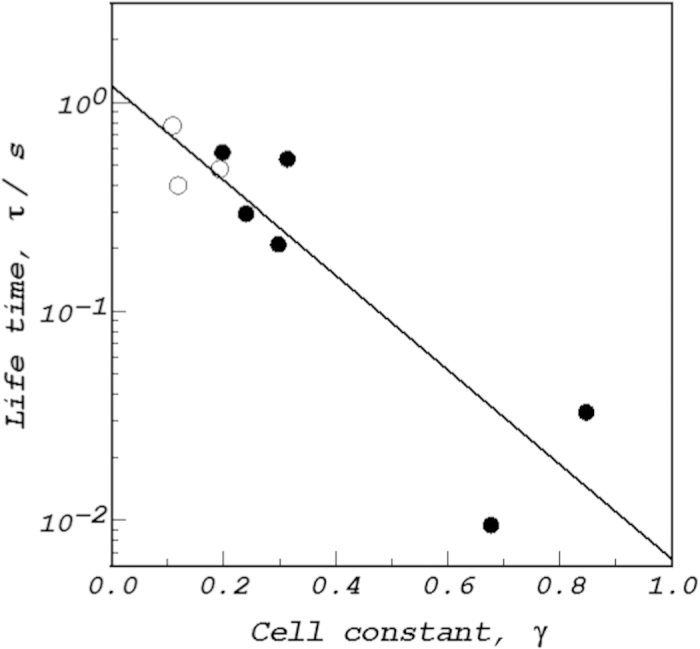
Plot of the lifetime of ionic vacancy vs. cell constant. ○, ferrocyanide oxidation and •, ferricyanide reduction in a 10^3^ mol m^−3^ KCl solution.

**Figure 7:**
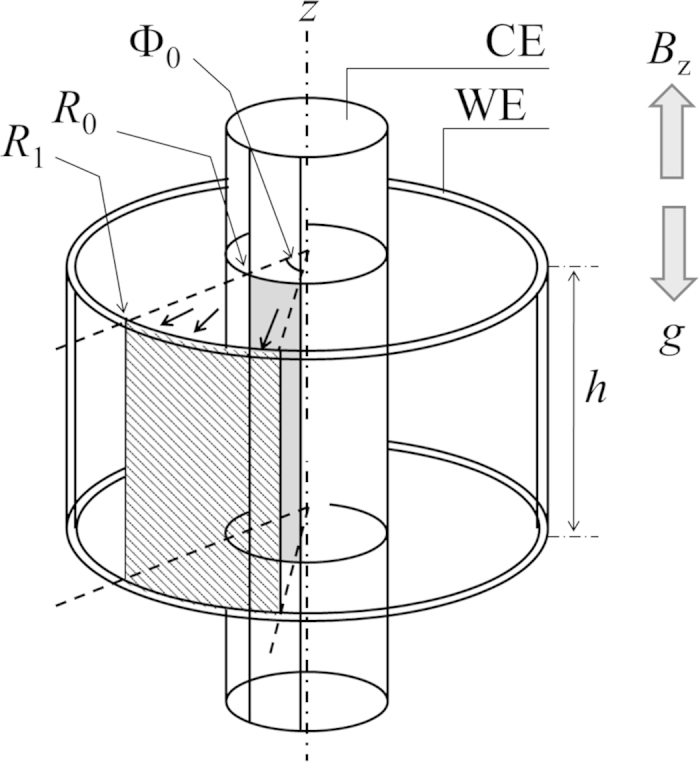
Schematic of a CMHDE with outer WE and inner CE. *R*_0_; inner radius, *R*_1_; outer radius, *h*; the electrode height, Φ_0_; the angle of the arc electrode surfaces, *B*_*z*_; the magnetic flux density, *g*; gravitational acceleration. Arrows indicate the directions of reduction current.

As shown in Fig. 4, the consumed amount of the reactant while coming through the plane 12 and leaving from the plane 34 is given by


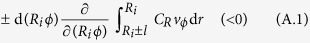


where the sign ± corresponds to *i* = 0 and 1 for WE, respectively. *v*_*ϕ*_ is defined positive. The mass transfer through the plane 24 compensates for the total mass loosing between the plane 12 and 34, i.e.,


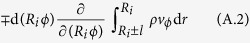


where the sign 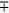 corresponds to *i* = 0 and 1, respectively. The amount of the reactant to participate the reaction, which is supplied from the plane 24, is also expressed by


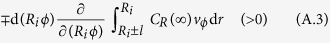


where *C*_*R*_(∞) is the bulk concentration. The reactant provided is consumed by the reaction at the plane 13, i.e., at WE. The amount of the reactant consumed at the electrode per unit time is expressed by


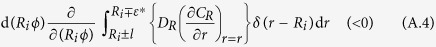


where *δ*(*r* − *R*_*i*_) is the *δ*-function, and *D*_*R*_ is the diffusion coefficient. The integration is performed between *R*_*i*_  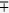 ε^*^ and *R*_*i  *_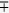 *l*, and passed to the limit ε^*^ = 0. The consuming rate of the reactant at the concentric element 1243 is


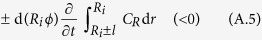


where the sign ± corresponds to *i* = 0 and 1, respectively. Using Eqs A.1, A.3, A.4 and A.5, we make the mass balance of the reactant, and then enlarge the concentric element to cover the electrode surface.


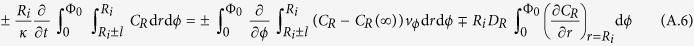


where *κ* is the stationary-mass-transfer coefficient introduced to express the initial non-steady diffusion.

For convenience, using the concentrations of the surface *C*_*R*_(*R*_*i*_) and the bulk *C*_*R*_(∞), we introduce the following parameters,


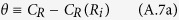



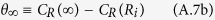


Equation A.6 is thus rewritten as


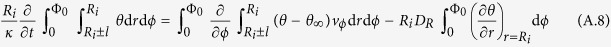


The boundary conditions of *θ* are as follows,


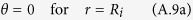



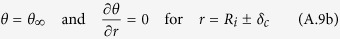


## Additional Information

**How to cite this article**: Sugiyama, A. *et al.* Lifetime of Ionic Vacancy Created in Redox Electrode Reaction Measured by Cyclotron MHD Electrode. *Sci. Rep.* **6**, 19795; doi: 10.1038/srep19795 (2016).

## Figures and Tables

**Figure 2:**
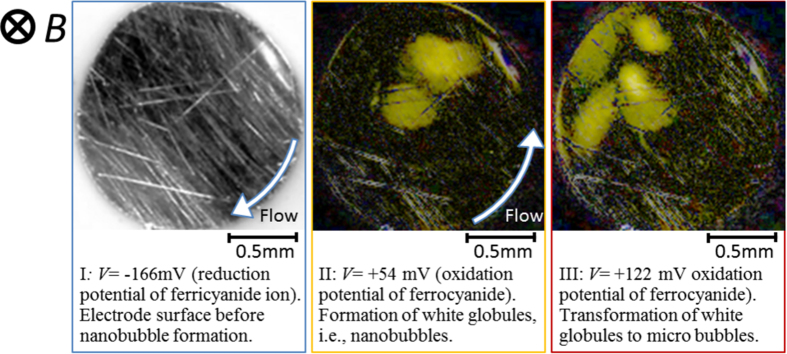
Mirobubble evolution on the electrode (newly refined^29^). I, Electrode surface during the reduction at an overpotential V = −166 mV (+264 mV vs. NHE); II, Nanobubble-layer formation with refractive variation at V = +37 mV (+467 mV vs. NHE). Accidental appearance of two globules of microbubble coalesced by microbubbles; III, Microbubble formation at V = +122 mV (+552 mV vs. NHE). Four globules newly formed. For visualization, the images are subtracted and painted by yellow.
